# Outcomes of Hospitalized COVID-19 Patients by Risk Factors: Results from a United States Hospital Claims Database

**DOI:** 10.36469/jheor.2020.17331

**Published:** 2020-09-29

**Authors:** Peter J. Mallow, Kathy W. Belk, Michael Topmiller, Edmond A. Hooker

**Affiliations:** 1 Xavier University, Department of Health Services Administration, Cincinnati, OH; 2 Health Clarity Solutions, LLC, Mooresville, NC; 3 HealthLandscape, LLC; American Academy of Family Physicians, Cincinnati, OH; 4 University of Cincinnati Medical Center, Cincinnati, OH

**Keywords:** COVID-19, health care utilization, mortality, outcomes, hospital, coronavirus

## Abstract

**Background/Objective:**

The primary objective was to quantify the role of the number of Centers of Disease Control and Prevention (CDC) risk factors on in-hospital mortality. The secondary objective was to assess the associated hospital length of stay (LOS), intensive care unit (ICU) bed utilization, and ICU LOS with the number of CDC risk factors.

**Methods:**

A retrospective cohort study consisting of all hospitalizations with a confirmed COVID-19 diagnosis discharged between March 15, 2020 and April 30, 2020 was conducted. Data was obtained from 276 acute care hospitals across the United States. Cohorts were identified based upon the number of the CDC COVID-19 risk factors. Multivariable regression modeling was performed to assess outcomes and utilization. The odds ratio (OR) and incidence rate ratio (IRR) were reported.

**Results:**

Compared with patients with no CDC risk factors, patients with risk factors were significantly more likely to die during the hospitalization: One risk factor (OR 2.08, 95% CI, 1.60–2.70; P < 0.001), two risk factors (OR 2.63, 95% CI, 2.00–3.47; P < 0.001), and three or more risk factors (OR 3.49, 95% CI, 2.53–4.80; P < 0.001). The presence of CDC risk factors was associated with increased ICU utilization, longer ICU LOS, and longer hospital LOS compared to those with no risk factors. Patients with hypertension (OR 0.77, 95% CI, 0.70–0.86; P < 0.001) and those administered statins were less likely to die (OR 0.54, 95% CI, 0.49–0.60; P < 0.001).

**Conclusions:**

Quantifying the role of CDC risk factors upon admission may improve risk stratification and identification of patients who may require closer monitoring and more intensive treatment.

## INTRODUCTION

The novel coronavirus disease 2019 (COVID-19) reached the United States in early 2020.^1^,^2^ It has since spread through the community and overwhelmed health care resources in several communities.^3^ Preliminary case studies and data reports have prompted the Centers for Disease Control and Prevention (CDC) to identify potential risk factors for COVID-19.^4^ These risk factors included advanced age (65 or older); residence in a long-term care/skilled nursing facility; and comorbid medical conditions including coronary artery disease, hypertension, chronic lung disease, moderate/severe asthma, obesity, diabetes, chronic kidney disease with dialysis, and other immunocompromising conditions (not specifically defined).^4^ Studies have shown most deaths from COVID-19 to be related to acute respiratory distress and other organ failure.^5^ However, the association of patient risk factors on in-hospital mortality and health care utilization for patients hospitalized with COVID-19 is uncertain.

Several studies have attempted to describe the presenting characteristics and associated hospital utilization by gender, age, and geography.^6^–^9^ These initial studies have described COVID-19 hospitalized mortality rates ranging from 5% to 64% based on age and lengths of stay varying from 4–13 days. These studies were limited to narrow geographic areas of New York City, New York; Toronto, Canada; and Wuhan, China. Furthermore, these studies did not examine the association of patient comorbidities on outcomes in the hospital setting.

The primary objective of this study was to quantify the role of the number of CDC risk factors on in-hospital mortality in a large and geographically diverse group of hospitalized COVID-19 patients. The secondary objective was to assess the associated hospital length of stay (LOS), intensive care unit (ICU) bed utilization, and ICU LOS with the number of CDC risk factors.

## DATA AND METHODS

### Data

Data were obtained retrospectively from a commercially-available, all-payer administrative database of inpatient and hospital-based outpatient detailed claims across more than 300 acute care hospitals in the US. Data contained information on demographic and clinical characteristics of all patient visits, including pharmaceuticals administered, diagnostic tests, and procedures performed during the hospitalization. Procedure and comorbidity data were captured using International Classification of Diseases 10th Revision (ICD-10) diagnosis and procedure codes. The research was conducted with a HIPAA compliant deidentified data set and was exempt from institutional review board review by Xavier University.

### Patient Visit Identification

COVID-19 patient visits between March 15, 2020 and April 30, 2020 were identified using ICD-10 diagnosis code U07.1. CDC risk factors associated with severe illness from COVID-19 were identified by ICD-10 codes with the exception of age (Table S1). Cholesterol and hypertension medications administered during the hospitalization were identified using hospital charge codes. A list of included statins, angiotensin-converting enzyme (ACE) inhibitors, and angiotensin II receptor blockers (ARBs) is found in Table S2. The Charlson Comorbidity Index was utilized to measure the burden of comorbid disease.^10^ Data did not permit the identification of race, ethnicity, or type of home setting (i.e. skilled nursing, long-term care) prior to admission. Patients were stratified into the following cohorts: zero, one, two, and three or more risk factors.

Data extracted included patient demographics (age, gender), comorbidities, diagnoses, procedures, discharge status, insurance, and medications administered during the hospital stay. Hospital characteristics included number of beds, geographic region, teaching status, and urban/rural location. Complications of interest included viral pneumonia, respiratory failure, sepsis, hyponatremia, hypernatremia, hypokalemia, hyperkalemia, and acidosis. Patient visit outcomes of interest were mortality, ICU bed utilization, ICU LOS, and hospital LOS.

### Statistical Analysis

Data were summarized with count and percentages for categorical data and mean and standard deviations for continuous data. Chi-square tests were used for categorical variables and analysis of variance for continuous variables to assess differences between comorbidity cohorts. Multivariable regression models were created for each outcome variable to examine the effect of the number of comorbidities. Logistic regression models were used for categorical outcomes and negative binomial regression were used to evaluate the adjusted incidence rate ratio (aIRR) for health care utilization outcomes. To adjust for confounding, model covariates included observed patient and treatment facility characteristics, including geographic region, teaching status, and bed size. A stepwise regression approach was used to include statistically significant patient and hospital characteristics. A patient’s Do-Not-Resuscitate (DNR) status and statin use in the hospital were also included as covariates.^11^–^13^ The CDC risk factor for chronic kidney disease (CKD) was limited to those undergoing dialysis. A sensitivity analysis including all CKD patients was performed. The odds ratio (OR) and aIRR were reported for categorical and continuous variables, respectively. A *P* value less than 0.05 was considered statistically significant. All significance testing was two-sided. All analyses were conducted in STATA (StataCorp, LLC, College Station, TX).

## RESULTS

A total of 21 676 hospitalizations with a COVD-19 diagnosis were identified across 276 hospitals in the database (Table 1). The average age of the patients was 64.9 and 52.8% were male. Nearly 23% of the patients died while hospitalized, 51% were discharged to home, and 15% were transferred to a long-term care/skilled nursing type facility. The remaining 11% were unknown discharge status, transferred to another hospital, or listed as other (Table 1).

The majority of hospitalized patients had two or more CDC risk factors (73.9%) with 44.3% having three or more CDC risk factors (Table 1). The average age was 43.1 for patients with no CDC risk factors and increased to 72.0 for those with three or more CDC risk factors (*P* < 0.001). The majority of patients with zero comorbid risk factors were male (57.9%), whereas there were slightly more females than males (50.5%) in patients with three or more CDC risk factors (*P* < 0.001). Patients with three or more CDC risk factors were more likely to have Medicare as a primary payer (70.1%; *P* < 0.001); however, 26% of this population had Medicaid as either a primary or secondary insurer.

Patients with zero or one CDC risk factors were discharged to home 80.5% and 64.3% of the time compared to 26.6% of patients with three or more CDC risk factors. In-hospital mortality increased significantly as the number of risk factors, including age, increased (Table 1; Table S3). Children (age less than 20) had extremely low mortality, with only one patient who had multiple comorbidities dying. The age group between 20 years and 49 years without comorbidities also had very low mortality, with only 1.9% dying. However, in all age groups, patients had significantly increased mortality if they had comorbidities. Patients between 40 and 59 years of age with three or more risk factors had a mortality rate near 20%. The mortality rate rose to more than 30% for patients age 70 to 89 and to 44% among patients 90 or older with three or more risk factors.

Hypertension was the most prevalent CDC risk factor in each of the cohorts (one CDC risk factor, 34.9%, to three or more, 90.4%; *P* < 0.001). Age, diabetes, and severe heart disease were also prevalent across all cohorts. Moderate to severe asthma was the least prevalent CDC risk factor across all cohorts (0% with one CDC risk factor to 0.9% with three or more; *P* < 0.001). The use of statins in the hospital ranged from 3.9% to 32.8% based on number of CDC risk factors (*P* < 0.001). ACE inhibitors and ARBs were administered in less than 9% of patients with three or more CDC risk factors.

The Charlson Comorbidity Index ranged from 0.08 to 3.83 for patients with zero to three or more CDC risk factors (*P* < 0.001). Dementia was present in 9.4% and 9.1% of hospitalized patients with two to three or more CDC risk factors, respectively. Hypothyroidism ranged from a low of 4.5% in patients with zero CDC risk factors to a high of 13% for patients with three or more risk factors (*P* < 0.001).

Patients were treated in hospitals located in all nine US census regions (Table 2). The highest concentration of patients were treated in hospitals located in the Mid-Atlantic region for all cohorts. The fewest number of patients were treated in hospitals located in the East South Central Region (Kentucky, Tennessee, Mississippi, and Alabama). Over 80% of the patients were treated in facilities located in urban areas across all cohorts. A majority of patients were seen at a teaching hospital (greater than 62% in all cohorts) and a plurality of patients were seen at a hospital with 500 or more beds (range 37.2% to 40.4%).

The top five pharmaceutical treatments provided during the hospitalization for those with two to three or more CDC risk factors were azithromycin, enoxaparin, hydroxychloroquine, zinc, and methylprednisolone (Table S4). Tocilizumab was used in 3.4% and 3.1% in those with zero or one CDC risk factors. Remdesivir was used in 0.5% or less of patients across the four cohorts.

The most prevalent complication during the hospitalization was viral pneumonia regardless of the number of CDC risk factors (Table 3). Respiratory failure ranged from 37.4% for those with no CDC risk factors to 56.9% for those with three or more (*P* < 0.001). Sepsis occurred in nearly 35% of all patients ranging from 20% of patients with zero CDC risk factors to 37.6% of those with three or more risk factors (*P* < 0.001). Hyponatremia and hypokalemia occurred in more than 11% of each cohort.

The mortality rate increased across the risk factor cohorts, ranging from 3.7% to 31.5% (*P* < 0.001). Patients with zero comorbid risk factors used the ICU 16.3% of the time with an average ICU LOS of 6.1 days. In contrast, patients with three or more CDC risk factors used the ICU 26.9% for an average ICU LOS of 7.8 days (*P* < 0.001). The average hospital LOS ranged from 6.8 to 9.7 days by cohort (*P* < 0.001).

Table 4 shows the multivariable regression results associated with mortality. Compared with patients with zero CDC risk factors, those with one risk factor (OR 2.08, 95% CI, 1.60–2.70; *P* < 0.001), two risk factors (OR 2.63, 95% CI, 2.00–3.47; *P* < 0.001), and three or more risk factors (OR 3.49, 95% CI, 2.53–4.80; *P* < 0.001) were significantly more likely to die during the hospitalization. Male patients were more likely to die in the hospital (OR 1.62; 95% CI, 1.50–1.75; *P* < 0.001) compared to females. Teaching hospitals were associated with fewer deaths (OR 0.91; 95% CI, 0.83–0.99; *P* < 0.001) compared to nonteaching hospitals. Patients with hypertension (OR 0.77; 95% CI, 0.70–0.86; *P* < 0.001) and statin use in the hospital (OR 0.54; 95% CI, 0.49–0.60; *P* < 0.001) were less likely to die.

The sensitivity analysis including all chronic kidney disease patients yielded similar results compared to the model using the CDC risk factor of CKD with dialysis (Table 4; Table S4). The use of ACE inhibitors and ARBs in the hospital was too low to be used reliably in the multivariable regression models.

Similar to mortality, the presence of more CDC risk factors was associated with increased ICU utilization, longer ICU LOS, and longer hospital LOS (Table 5). The full multivariable regression results can be found in Table S5. Compared to patients with zero CDC risk factors, patients with three or more risk factors were more likely to require an ICU bed (OR 2.18; 95% CI, 1.72–2.76; *P* < 0.001), stay longer in the ICU (OR 1.34; 95% CI, 1.14–1.58; *P* < 0.001), and stay longer in the hospital (OR 1.35; 95% CI, 1.26–1.44; *P* < 0.001).

## DISCUSSION

The present study of more than 20 000 COVID-19 hospitalizations across the United States found that patients with three or more CDC risk factors were associated with a nearly 4.5 times increase in mortality. Further, it confirms that diabetes, obesity, and CKD with dialysis were critical risk factors with respect to individuals requiring increased care. Significant increases in ICU bed utilization, longer ICU stay, and longer hospital LOS were associated with the presence of three or more risk factors.

While patients with three or more risk factors had the highest risk of mortality and increased health care utilization, those with one or two risk factors were more than three times as likely to die in the hospital compared to those with no risk factors. Similarly, patients hospitalized with one or two risk factors were more than two times as likely to require an ICU bed and remain in the ICU and hospital longer. Our findings confirm and begin to quantify the role of the CDC risk factors regarding the potential for a severe and extended hospitalization.

Our results did not find a statistically significant association of patients with moderate to severe asthma or immunocompromised status with mortality. However, we caution that the small sample size for asthma and narrow definition for immunocompromised status may be influencing the results. The CDC’s guidance for immunocompromised status was vague. Our definition included cancer, autoimmune diseases, and HIV/AIDS. Further research is necessary to assess the risk of immunocompromised status of a broader range of comorbid conditions.

With respect to CKD we found that broadening the definition beyond patients requiring dialysis yielded similar results suggesting all patients with CKD may be at an increased risk.

Of particular interest is the decreased likelihood of in-hospital mortality with statin use. Our findings suggest that patients administered statins in the hospital had a 46% lower risk of death than those not receiving statins. Caution in interpretation of the association between statin use and mortality is warranted as our data did not include frequency of administration or dose while in the hospital. However, this finding is similar to emerging research suggesting statin use may be a low-cost adjuvant therapy, though the specific mechanism of action is not yet defined.^11^,^12^ Unfortunately, the low use of in-hospital administration of ACE inhibitors and ARBs in this population prevented the exploration of their association with mortality.

Similar to mortality, patients with one or more risk factors were more likely to require an ICU bed and stay longer in the ICU and hospital. Our results provide evidence to support the triaging of patients based on the published CDC risk factors for severity of COVID-19. Despite differing definitions, our study compared favorably with a study examining comorbid risk factors in China.^14^ This study examined 1590 COVID-19 hospitalized patients in China and found 10 independent predictors associated with severe illness. Patients with one or more risk factors were more likely to have poor outcomes (OR 1.60). Our findings combined with Liang et al. (2020) and Popkin et al. (2020) provide further evidence that a detailed history and identification of comorbid conditions during triage may allow for risk stratification and identification of patients who may benefit from increased monitoring and interventions.^14^,^15^

We examined the CDC listed risk factors as a starting point.^4^ These risk factors were largely consistent with those reported previously.^6^–^9^,^14^ However, we recognize that there may be additional risk factors not correlated with the CDC risk factors or variables included in the Charlson Comorbidity Index. For example, one in six patients with no CDC risk factors required an ICU stay. Additionally, we did not examine the relationship between age independently from other comorbid conditions. We observed a greater number of comorbidities in the older population. It is unknown whether age itself is a driving factor of increased risk or the increased presence of comorbidities in the aging population. Further exploration of the secondary diagnoses, in particular in those under the age of 50, is warranted. Furthermore, additional examination of the impact of statin administration on the outcomes of patients with COVID-19 should be considered.

### Limitations

Our study and the data used were not without limitations. The Mid-Atlantic region (includes New York and New Jersey) has been overwhelmed by COVID-19 cases and accounted for 47% of our patients. Further research is necessary to explore the role of geographic factors. Second, the use of claims data may under-report the number of COVID-19 related hospitalizations. For example, we did not include probable COVID-19 hospitalizations. The CDC has not provided a specific list of immunocompromised conditions. We used a narrow definition of immunocompromised status that may underestimate the true number of comorbidities per patient. The use of statins, ACE inhibitors, and ARBs was limited to in-hospital use identified through the hospital chargemaster. We did not have data on the frequency or dose when administered. As with all observational studies, issues of collinearity and overfitting may be present. Our results should be interpreted as correlations rather than causal. However, our results combined with similar studies with other data sources and study designs strengthen the associations witnessed in this fast-evolving pandemic. The data did not include a patient’s race or ethnicity. Research is emerging suggesting race and ethnicity are associated with a substantial increase in death from COVID-19.^16^ Finally, the use of retrospective data intended to capture information for financial accounting may omit relevant clinical data creating an under- or over-estimate of the adjusted results. However, the use of claims data remains an important source when investigating hospital outcomes and utilization.^17^,^18^ Despite these limitations, this study provides further evidence of the effect of comorbidities on in-hospital outcomes related to the novel coronavirus epidemic.

## CONCLUSION

In this sample of US hospitals, patients presenting with any number of CDC risk factors fared poorly compared to those without. Our findings suggest that patients with three or more risk factors were more than 4.5 times as likely to die during the hospitalization. Further, they were more likely to require an ICU stay and longer LOS in the ICU and hospital. Those on statins in the hospital were nearly 50% less likely to die. Further investigation into this association between statin usage and mortality is warranted. A thorough patient history and comorbid assessment may allow for better risk stratification upon admission perhaps enabling a better use of resources in the event of hospital capacity limits during a strong second wave of the pandemic.

## Supplementary Information



## Figures and Tables

**Table 1:** COVID-19 Patient Characteristics by Number of Risk Factors

	Total Population		Zero Risk Factors		One Risk Factor		Two Risk Factors		Three or More Risk Factors		*P* Value
	Count	%	Count	%	Count	%	Count	%	Count	%	
Total	21 676	100	2227	10.3%	3424	15.8%	6432	29.7%	9593	44.3%	
**Patient Age**											
0–19	121	0.6%	69	3.1%	36	1.1%	11	0.2%	5	0.1%	
20–29	588	2.7%	289	13.0%	175	5.1%	82	1.3%	42	0.4%	
30–39	1256	5.8%	511	22.9%	366	10.7%	224	3.5%	155	1.6%	
40–49	2045	9.4%	529	23.8%	616	18.0%	517	8.0%	383	4.0%	
50–59	3540	16.3%	599	26.9%	980	28.6%	1151	17.9%	810	8.4%	
60–69	4744	21.9%	230	10.3%	816	23.8%	1456	22.6%	2242	23.4%	
70–79	4399	20.3%	0	0.0%	213	6.2%	1125	17.5%	3061	31.9%	
80–89	3525	16.3%	0	0.0%	154	4.5%	1188	18.5%	2183	22.8%	
90+	1458	6.7%	0	0.0%	68	2.0%	678	10.5%	712	7.4%	<.001
*Age in years (mean/std dev)*	*64.9*	*17.2*	*43.1*	*13.2*	*54.1*	*15.3*	*67.6*	*16.0*	*72.0*	*12.7*	<.001
**Gender**											
Female	10 234	47.2%	937	42.1%	1477	43.1%	2979	46.3%	4841	50.5%	
Male	11 442	52.8%	1290	57.9%	1947	56.9%	3453	53.7%	4752	49.5%	<.001
**Discharge Status**											
Expired	4936	22.8%	83	3.7%	387	11.3%	1446	22.5%	3020	31.5%	
Home	9080	41.9%	1793	80.5%	2202	64.3%	2532	39.4%	2553	26.6%	
Home health	2001	9.2%	97	4.4%	240	7.0%	603	9.4%	1061	11.1%	
Hospice	729	3.4%	8	0.4%	54	1.6%	272	4.2%	395	4.1%	
LTC	231	1.1%	9	0.4%	19	0.6%	82	1.3%	121	1.3%	
Other	362	1.7%	68	3.1%	71	2.1%	107	1.7%	116	1.2%	
Rehab	426	2.0%	17	0.8%	58	1.7%	132	2.1%	219	2.3%	
SNF or ICF	2571	11.9%	43	1.9%	183	5.3%	814	12.7%	1531	16.0%	
Transfer	708	3.3%	76	3.4%	119	3.5%	229	3.6%	284	3.0%	
Missing/unknown	632	2.9%	33	1.5%	91	2.7%	215	3.3%	293	3.1%	<.001
**Primary Payer**											
Commercial	5752	26.5%	912	41.0%	1420	41.5%	1743	27.1%	1677	17.5%	
Medicaid	2772	12.8%	603	27.1%	673	19.7%	755	11.7%	741	7.7%	
Medicare	10 889	50.2%	159	7.1%	716	20.9%	3291	51.2%	6723	70.1%	
Other	1142	5.3%	251	11.3%	314	9.2%	315	4.9%	262	2.7%	
Missing/unknown	1121	5.2%	302	13.6%	301	8.8%	328	5.1%	190	2.0%	<.001
**Medicaid**											
Medicaid as any payer	5700	26.3%	701	31.5%	952	27.8%	1553	24.1%	2494	26.0%	<.001
**Statin/ACE/ARBs Medications Administered in Hospital**											
Statins	5313	24.5%	87	3.9%	425	12.4%	1659	25.8%	3142	32.85%	<.001
ACE inhibitors	1472	6.8%	5	0.2%	127	3.7%	513	8.0%	827	8.6%	<.001
ARBs	1277	5.9%	4	0.2%	100	2.9%	405	6.3%	768	8.0%	<.001
**CDC Comorbid Risk Factors**											
Age >65	11 695	54.0%	0	0.0%	600	17.5%	3564	55.4%	7531	78.5%	<.001
Chronic lung disease	4654	21.5%	0	0.0%	336	9.8%	613	9.5%	3705	38.6%	<.001
Moderate to severe asthma	100	0.5%	0	0.0%	0	0.0%	14	0.2%	86	0.9%	<.001
Severe heart disease	12 000	55.4%	0	0.0%	756	22.1%	3854	59.9%	7390	77.0%	<.001
Immunocompromised	1997	9.2%	0	0.0%	155	4.5%	314	4.9%	1528	15.9%	<.001
Obesity	3029	14.0%	0	0.0%	346	10.1%	654	10.2%	2029	21.2%	<.001
Diabetes	9167	42.3%	0	0.0%	393	11.5%	1765	27.4%	7009	73.1%	<.001
CKD with dialysis	1269	5.9%	0	0.0%	8	0.2%	113	1.8%	1148	12.0%	<.001
Liver disease	936	4.3%	0	0.0%	88	2.6%	179	2.8%	669	7.0%	<.001
Hypertension	14 757	68.1%	0	0.0%	1195	34.9%	4887	76.0%	8675	90.4%	<.001
**Other Comorbidities**											
CKD (any stage)	1470	6.8%	28	1.3%	181	5.3%	113	1.8%	1148	12.0%	<.001
Hemoptysis	164	0.8%	14	0.6%	31	0.9%	46	0.7%	73	0.8%	0.649
Hypothyroidism	2260	10.4%	101	4.5%	223	6.5%	688	10.7%	1248	13.0%	<.001
DNR Status	5301	24.5%	59	2.6%	342	10.0%	1775	27.6%	3125	32.6%	<.001
**Charlson Comorbidities**											
Myocardial infarction	1780	8.2%	0	0.0%	83	2.4%	504	7.8%	1193	12.4%	<.001
Congestive heart failure	3479	16.1%	0	0.0%	92	2.7%	786	12.2%	2601	27.1%	<.001
Peripheral vascular disease	1030	4.8%	5	0.2%	56	1.6%	288	4.5%	681	7.1%	<.001
Cerebrovascular disease	1547	7.1%	26	1.2%	118	3.4%	470	7.3%	933	9.7%	<.001
Dementia	1659	7.7%	26	1.2%	154	4.5%	604	9.4%	875	9.1%	<.001
Chronic pulmonary disease	4654	21.5%	0	0.0%	336	9.8%	613	9.5%	3705	38.6%	<.001
Connective tissue disease	489	2.3%	7	0.3%	34	1.0%	105	1.6%	343	3.6%	<.001
Peptic ulcer disease	160	0.7%	9	0.4%	10	0.3%	36	0.6%	105	1.1%	<.001
Mild liver disease	247	1.1%	0	0.0%	20	0.6%	40	0.6%	187	1.9%	<.001
Diabetes without end-organ damage	5057	23.3%	0	0.0%	248	7.2%	1073	16.7%	3736	38.9%	<.001
Diabetes with end-organ damage	3930	18.1%	0	0.0%	40	1.2%	397	6.2%	3493	36.4%	<.001
Hemiplegia	277	1.3%	17	0.8%	46	1.3%	83	1.3%	131	1.4%	0.146
Moderate or severe renal disease	5360	24.7%	33	1.5%	208	6.1%	1269	19.7%	3850	40.1%	<.001
Tumor without metastases	959	4.4%	0	0.0%	55	1.6%	116	1.8%	788	8.2%	<.001
Moderate or severe liver disease	155	0.7%	0	0.0%	12	0.4%	34	0.5%	109	1.1%	<.001
Metastatic solid tumor	265	1.2%	0	0.0%	23	0.7%	38	0.6%	204	2.1%	<.001
AIDS	149	0.7%	0	0.0%	20	0.6%	27	0.4%	102	1.1%	<.001
*Charlson Comorbidity Index (mean/sd)*	*2.3*	*2.5*	*0.1*	*0.4*	*0.7*	*1.3*	*1.5*	*1.7*	*3.8*	*2.6*	<.001

Abbreviations: ACE, angiotensin-converting enzyme; ARB, angiotensin II receptor blockers; CDC, Centers for Disease Control and Prevention; HTN, hypertension; ICF, intermediate care facility; LTC, long-term care facility; SNF, skilled nursing facility.

**Table 2:** Hospital Characteristics of COVID-19 Patient Visits

	Zero Risk Factors		One Risk Factor		Two Risk Factors		Three or More Risk Factors		*P* Value
	Count	%	Count	%	Count	%	Count	%	
Total	2227	10.3%	3424	15.8%	6432	29.7%	9593	44.3%	
**Bed Size**									
Less than 100	81	3.6%	129	3.8%	252	3.9%	355	3.7%	
100–199	308	13.8%	540	15.8%	1076	16.7%	1544	16.1%	
200–299	400	18.0%	640	18.7%	1250	19.4%	1745	18.2%	
300–499	536	24.1%	724	21.1%	1459	22.7%	2076	21.6%	
500 or more	902	40.5%	1391	40.6%	2395	37.2%	3873	40.4%	0.001
**Teaching Status**									
Teaching	1386	62.2%	2174	63.5%	4067	63.2%	6203	64.7%	
Nonteaching	841	37.8%	1250	36.5%	2365	36.8%	3390	35.3%	<.001
**Region**									
East North Central	62	2.8%	135	3.9%	249	3.9%	425	4.4%	
East South Central	5	0.2%	19	0.6%	32	0.5%	46	0.5%	
Middle Atlantic	1154	51.8%	1646	48.1%	2953	45.9%	4419	46.1%	
Mountain	146	6.6%	183	5.3%	257	4.0%	381	4.0%	
New England	194	8.7%	332	9.7%	693	10.8%	962	10.0%	
Pacific	138	6.2%	179	5.2%	352	5.5%	460	4.8%	
South Atlantic	295	13.2%	449	13.1%	822	12.8%	1197	12.5%	
West North Central	33	1.5%	54	1.6%	64	1.0%	125	1.3%	
West South Central	200	9.0%	427	12.5%	1010	15.7%	1578	16.4%	<.001
**Urban/Rural Location**									
Rural	369	16.6%	548	16.0%	959	14.9%	1808	18.8%	
Urban	1858	83.4%	2876	84.0%	5473	85.1%	7785	81.2%	<.001

**Table 3:** COVID-19 Patient Complications and Outcomes by Number of Risk Factors

	Total Population		Zero Risk Factors		One Risk Factor		Two Risk Factors		Three or More Risk Factors		*P* Value
	Count	%	Count	%	Count	%	Count	%	Count	%	
**Complications of Disease**											
Viral pneumonia	17 768	82.0%	1581	71.0%	2785	81.3%	5335	82.9%	8067	84.1%	<.001
Respiratory failure	11 130	51.3%	834	37.4%	1577	46.1%	3263	50.7%	5456	56.9%	<.001
Acute kidney failure	7705	35.5%	177	7.9%	711	20.8%	2437	37.9%	4380	45.7%	<.001
Sepsis	7440	34.3%	505	22.7%	1008	29.4%	2319	36.1%	3608	37.6%	<.001
Hyponatremia	3817	17.6%	320	14.4%	542	15.8%	1177	18.3%	1778	18.5%	<.001
Acidosis	3117	14.4%	109	4.9%	325	9.5%	920	14.3%	1763	18.4%	<.001
Hyperkalemia	2867	13.2%	63	2.8%	208	6.1%	630	9.8%	1572	16.4%	<.001
Hypernatremia	2473	11.4%	60	2.7%	293	8.6%	1006	15.6%	1508	15.7%	<.001
Hypokalemia	3414	15.8%	261	11.7%	554	16.2%	1134	17.6%	1465	15.3%	<.001
**Outcomes**											
Mortality	4936	22.8%	83	3.7%	387	11.3%	1446	22.5%	3020	31.5%	<.001
ICU during stay	5250	24.2%	363	16.3%	784	22.9%	1522	23.7%	2581	26.9%	<.001
*ICU Days (mean/sd)*	*7.6*	*6.8*	*6.1*	*5.8*	*7.0*	*6.5*	*7.8*	*6.8*	*7.8*	*7.0*	<.001
*Hospital LOS (mean/std)*	*8.9*	*7.3*	*6.8*	*7.8*	*8.1*	*6.4*	*8.9*	*6.6*	*9.7*	*7.9*	<.001

**Table 4:** Association of Risk Factors with Mortality

	Odds Ratio	95% Confidence Interval		*P* Value
**CDC Risk Factor Cohorts**				
0	Reference			
1	2.08	1.60	2.70	<0.001
2	2.63	2.00	3.47	<0.001
3 or more risk factors	3.49	2.53	4.80	<0.001
Age	1.02	1.02	1.02	<0.001
**Gender**				
Female	Reference			
Male	1.62	1.50	1.75	<0.001
**Insurance**				
Medicaid as any payer	1.10	1.01	1.20	0.036
**Teaching Status**				
Nonteaching hospital	Reference			
Teaching hospital	0.91	0.83	0.99	0.032
**Hospital Bed Size**				
0–99	Reference			
100–199	1.71	1.33	2.21	<0.001
200–299	1.74	1.35	2.23	<0.001
300–499	2.19	1.70	2.82	<0.001
500 or more	2.22	1.72	2.85	<0.001
**CDC Risk Factors**				
Chronic lung disease	0.89	0.80	0.98	0.023
Moderate to severe asthma	1.10	0.60	2.04	0.754
Heart condition	1.27	1.16	1.40	<0.001
Immunocompromised	0.89	0.78	1.01	0.073
Obesity	1.30	1.15	1.47	<0.001
Diabetes	1.30	1.17	1.44	<0.001
CKD with dialysis	1.46	1.26	1.70	<0.001
Liver disease	1.91	1.61	2.26	<0.001
Hypertension	0.77	0.70	0.86	<0.001
DNR	9.01	8.27	9.81	<0.001
Statin use in hospital	0.54	0.49	0.60	<0.001

Abbreviation: CDC, Centers for Disease Control and Prevention.

**Table 5:** Association of Risk Factors with ICU Utilization, ICU Length of Stay, and Hospital Length of Stay

ICU Utilization	Odds Ratio	95% Confidence Interval		*P* Value
**CDC Risk Factor Cohorts**				
0	Reference			
1	1.68	1.44	1.97	<0.001
2	1.80	1.50	2.16	<0.001
3 or more risk factors	2.18	1.72	2.76	<0.001
**ICU Length of stay**	**aIRR**	**95% Confidence Interval**		***P* Value**
**CDC Risk Factor Cohorts**				
0	Reference			
1	1.15	1.03	1.28	0.016
2	1.29	1.14	1.46	<0.001
3 or more risk factors	1.34	1.14	1.58	<0.001
**Hospital Length of Stay**	**aIRR**	**95% Confidence Interval**		***P* Value**
**CDC Risk Factor Cohorts**				
0	Reference			
1	1.16	1.12	1.21	<0.001
2	1.26	1.20	1.33	<0.001
3 or more risk factors	1.35	1.26	1.44	<0.001

Abbreviation: aIRR, adjusted incidence rate ratio; CDC, Centers for Disease Control and Prevention.
