# Supplementary material for: Outcomes of Hospitalized COVID-19 Patients by Risk Factors: Results from a United States Hospital Claims Database
Source: J Health Econ Outcomes Res. 2020 Sep 29;7(2):165–74. doi: 10.36469/jheor.2020.17331 (PMC7539762; doi:10.36469/jheor.2020.17331)
Supplement: Supplementary file 1 [file jheor-7-2-17331-s01.pdf]

Journal of Health Economics and Outcomes Research

Supplementary Online Material

Mallow PJ, Belk KW, Topmiller M, Hooker EA. Outcomes of hospitalized COVID-19 patients by risk factors: Results from a United States hospital claims database. *JHEOR*. 2020;7(2):165-174.
doi:10.36469/jheor.2020.17331

Table S1. ICD-10 Codes Used to Identify Comorbid Conditions Contributing to Risk Factors
Table S2. Cholesterol/Hypertension Medications Administered at Hospital
Table S3. Mortality by Age Range and Risk Factors
Table S4. Hospital Treatments by Number of Risk Factors
Table S5. Association of Risk Factors with Mortality with Broad Renal Disease Definition, ICU Utilization, ICU Length of Stay, and Hospital Length of Stay: Complete Model data

This supplementary material has been provided by the authors to give readers additional information about their work.

This is an open-access article distributed under the terms of the Creative Commons Attribution 4.0 International License (CCBY-4.0). View this license's legal deed at http://creativecommons.org/licenses/by/4.0 and legal code at http://creativecommons.org/licenses/by/4.0/legalcode for more information.

Table S1. ICD-10 Codes Used to Identify Comorbid Conditions Contributing to Risk Factors

Chronic Lung Disease							
J40.x	J41.x	J42.x	J43.x	J44.x	J45.x	J46.x	J47.x
Moderate-to Severe Asthma							
J45.4	J45.5						
Heart Condition							
I21.x	I22.x	I20.0x	I50.x	I25.x	I48.x	E78.x	
Immunocompromised							
C00-D49.99	K58.x	M05.x	M06.x	L40.x	M45.x	B20.x	
Obesity							
E66.01	E66.2	Z68.35	Z68.36	Z68.37	Z68.38	Z68.39	Z68.4x
Diabetes							
E08.x	E09.x	E10.x	E11.x	E12.x	E13.x		
CKD with Dialysis							
N18.5	N18.6						
Liver Disease							
K70.x	K71.x	K72.x	K73.x	K74.x	K75.x	K76.x	K77.x
Hypertension							
I10.x	I11.x	I12.x	I13.x	I14.x	I15.x	I6.x	

Table S2. Cholesterol/Hypertension Medications Administered at Hospital

Statins	Statins (cont.)	Statins (cont.)	Statins (cont.)	ACE Inhibitors	ACE Inhibitors (cont.)	ACE Inhibitors (cont.)	ARBs
Advicor	Ezallor	pitavastatin	Sinvalip	Accupril	Listril	Tritace	almesartan
Alipza	Flolipid	Prasterol	Sinvat	Aceon	Lopril	Univasc	atacand
Alpheus	fluvastatin	Pravachol	Sivastin	Alacepril	Lotensin	Vasotec	avapro
Altocor	Goltor	Pravaselect	Sortis	Altace	Mavik	Zefnopril	azilsartan
Altoprev	Inegy	pravastatin	Staros	Arfalasin	Moexipril	Zestril	benicar
Aplactin	Koleros	Primesin	Staticol	Benazepril	Monopril		byvalson
Arkas	Krustat	Provisacor	Torvacard	Berlipril	Novatec		candesartan
Atocor	Lescol	Rosastin	Torvast	Capoten	Odrik		cozaar
Ator	Lipenil	Rosim	Totalip	Captopril	Perindo		diovan
Atoris	Lipertance	Rosulip	Triveram	Cilazapril	Perindopril		edarbi
Atorlip	Lipex	Rosumibe	Vastgen	Coversyl	Prilace		entresto
atorvastatin	Lipidover	rosuvastatin	Vasticor	Enalapril	Prinivil		eprosartan
Baycol	Lipitor	Sanaprav	Vastin	Enalapril	Profarma		irbesartan
Caduet	Liponorm	Selectin	Viazet	Enap	Qbrelis		losartan
Canef	Lipostat	Selektine	Vytorin	Epaned	Quinapril		micardis
cerivastatin	Livalo	Setorlin	Xipocol	Fosinopril	Ramace		prexxartan
Cholecomb	Livazo	Simbatrix	Zestan	Fosinopril	Ramipril		telmisartan
Colcardiol	lovastatin	Simcard	Zevistat	Fositen	ramipril		tevetan
Colfri	Medipo	Simcor	Zocor	Gopten	Ramitac		valsartan
Compactin	Mevacor	Simcora	Zypitamag	Imidapril	Ramiwin		
Crestor	Miastina	Simestat		Inhibace	Renitec		
Delipid	Nikita	simvastatin		Lexxel	Tanatril		
Dilivas	Omistat	Sincol		Lisidigal	Trandolapril		
Exorta	Pitava	Sinvacor		Lisinopril	Triatec		

Abbreviations: ACE, angiotensin-converting enzyme; ARB, angiotensin II receptor blockers.

Table S3. Mortality by Age Range and Risk Factors

	Zero Risk Factors			One Risk Factor			Two Risk Factors			Three or More Risk Factors			*P* Value[a]
Age	Pop.	Mortality	Mortality Rate	Pop.	Mortality	Mortality Rate	Pop.	Mortality	Mortality Rate	Pop.	Mortality	Mortality Rate	
0–19	69	0	0.0%	36	0	0.0%	11	1	9.1%	5	0	0.0%	0.132
20–29	289	4	1.4%	175	3	1.7%	82	7	8.5%	42	3	7.1%	0.002
30–39	511	8	1.6%	366	17	4.6%	224	17	7.6%	155	15	9.7%	<.001
40–49	529	14	2.6%	616	27	4.4%	517	44	8.5%	383	72	18.8%	<.001
50–59	599	35	5.8%	980	84	8.6%	1151	153	13.3%	810	166	20.5%	<.001
60–69	230	22	9.6%	816	100	12.3%	1456	241	16.6%	2242	546	24.4%	<.001
70–79	0	0	0.0%	213	58	27.2%	1125	295	26.2%	3061	991	32.4%	<.001
80–89	0	0	0.0%	154	68	44.2%	1188	443	37.3%	2183	914	41.9%	0.022
90+	0	0	0.0%	68	29	42.6%	678	245	36.1%	712	313	44.0%	0.01

[a]Comparing Risk Factors for Each Age Group groups for each age group

Table S4. Hospital Treatments by Number of Risk Factors

	Zero Risk Factors		One Risk Factor		Two Risk Factors		Three or More Risk Factors	
	**Count**	**%**	**Count**	**%**	**Count**	**%**	**Count**	**%**
Total	2885	10.5%	4686	17.0%	8442	30.7%	11471	41.7%
**Treatments**								
azithromycin	898	31.1%	1539	32.8%	2797	33.1%	3912	34.1%
enoxaparin	925	32.1%	1517	32.4%	2534	30.0%	3099	27.0%
hydroxychloroquine	721	25.0%	1168	24.9%	2210	26.2%	3097	27.0%
zinc	341	11.8%	462	9.9%	901	10.7%	1363	11.9%
methylprednisolone	95	3.3%	202	4.3%	426	5.0%	742	6.5%
apixaban	37	1.3%	93	2.0%	331	3.9%	735	6.4%
clopidogrel	2	0.1%	36	0.8%	249	2.9%	561	4.9%
alteplase	22	0.8%	74	1.6%	171	2.0%	319	2.8%
tocilizumab	98	3.4%	145	3.1%	256	3.0%	313	2.7%
nifedipine	5	0.2%	33	0.7%	89	1.1%	205	1.8%
acetazolamide	10	0.3%	36	0.8%	73	0.9%	127	1.1%
rivaroxaban	11	0.4%	36	0.8%	104	1.2%	104	0.9%
ritonavir	3	0.1%	11	0.2%	19	0.2%	28	0.2%
remdesivir	15	0.5%	24	0.5%	30	0.4%	28	0.2%
argatroban	1	0.0%	6	0.1%	13	0.2%	27	0.2%
lopinavir ritonavir	2	0.1%	8	0.2%	16	0.2%	24	0.2%
cyclosporine	0	0.0%	3	0.1%	9	0.1%	20	0.2%
darunavir	3	0.1%	12	0.3%	11	0.1%	17	0.1%
Ivermectin	3	0.1%	9	0.2%	16	0.2%	17	0.1%
dabigatran	1	0.0%	2	0.0%	7	0.1%	16	0.1%
fondaparinux	2	0.1%	3	0.1%	13	0.2%	15	0.1%
tranexamic acid	8	0.3%	2	0.0%	7	0.1%	15	0.1%
bivalirudin	1	0.0%	2	0.0%	2	0.0%	10	0.1%
darunavir cobicistat	1	0.0%	7	0.1%	3	0.0%	9	0.1%
chloroquine	0	0.0%	0	0.0%	0	0.0%	3	0.0%
atazanavir	1	0.0%	0	0.0%	5	0.1%	3	0.0%
canagliflozin	0	0.0%	0	0.0%	1	0.0%	3	0.0%
nitazoxanide	3	0.1%	2	0.0%	2	0.0%	3	0.0%
ddoxaban	0	0.0%	0	0.0%	0	0.0%	1	0.0%
fosamprenavir	0	0.0%	0	0.0%	0	0.0%	1	0.0%
tenecteplase	0	0.0%	1	0.0%	2	0.0%	1	0.0%

Table S5. Association of Risk Factors with Mortality with Broad Renal Disease Definition, ICU Utilization, ICU Length of Stay, and Hospital Length of Stay: Complete Model data

Mortality w/ Broad Renal Disease Definition	Odds Ratio	95% Confidence Interval		*P* Value
**CDC Risk Factor Cohorts**				
0	Reference			
1	2.16	1.66	2.80	<0.001
2	2.76	2.10	3.64	<0.001
3 or More Risk Factors	3.68	2.68	5.06	<0.001
**Age**	1.02	1.01	1.02	<0.001
**Gender**				
Female	Reference			
Male	1.58	1.47	1.71	<0.001
**Insurance**				
Medicaid as Any Payer	1.10	1.01	1.20	0.036
**Teaching Status**				
Nonteaching Hospital	Reference			
Teaching Hospital	0.90	0.82	0.99	0.026
**Hospital Bed Size**				
0–99	Reference			
100–199	1.75	1.36	2.26	<0.001
200–299	1.78	1.39	2.30	<0.001
300–499	2.24	1.74	2.88	<0.001
500 or More	2.26	1.76	2.91	<0.001
**Comorbid Risk Factors**				
Chronic Lung Disease	0.87	0.78	0.96	0.007
Moderate-to-Severe Asthma	1.11	0.60	2.05	0.747
Heart Condition	1.24	1.13	1.36	<0.001
Immunocompromised	0.87	0.76	0.99	0.035
Obesity	1.26	1.11	1.42	<0.001
Diabetes	1.24	1.12	1.37	<0.001
Renal Disease	1.56	1.43	1.70	<0.001
Liver Disease	1.91	1.61	2.26	<0.001
Hypertension	0.74	0.66	0.82	<0.001
DNR	8.90	8.17	9.70	<0.001
Statin Use in Hospital	0.54	0.49	0.59	<0.001
**ICU Utilization**	**Odds Ratio**	**95% Confidence Interval**		***P* Value**
**CDC Risk Factor Cohorts**				
0	Reference			
1	1.68	1.44	1.97	<0.001
2	1.80	1.50	2.16	<0.001
3 or More Risk Factors	2.18	1.72	2.76	<0.001
**Age**	0.98	0.98	0.99	<0.001

Table S5. Association of Risk Factors with Mortality with Broad Renal Disease Definition, ICU Utilization, ICU Length of Stay, and Hospital Length of Stay: Complete Model data

**Gender**				
Female	Reference			
Male	1.31	1.22	1.40	<0.001
**Insurance**				
Medicaid as Any Payer	0.76	0.70	0.82	<0.001
**Teaching Status**				
Nonteaching Hospital	Reference			
Teaching Hospital	0.42	0.39	0.45	<0.001
**Hospital Bed Size**				
0–99	Reference			
100–199	0.92	0.78	1.10	0.365
200–299	1.01	0.85	1.19	0.909
300–499	1.36	1.15	1.60	<0.001
500 or More	0.82	0.69	0.97	0.019
**Comorbid Risk Factors**				
Chronic Lung Disease	0.88	0.80	0.96	0.006
Moderate-to-Severe Asthma	0.97	0.60	1.55	0.885
Heart Condition	1.13	1.04	1.23	0.004
Immunocompromised	0.88	0.78	1.00	0.046
Obesity	1.37	1.23	1.52	<0.001
Diabetes	1.09	1.00	1.20	0.056
Renal Disease	0.95	0.82	1.10	0.505
Liver Disease	1.67	1.44	1.94	<0.001
Hypertension	0.85	0.77	0.93	<0.001
DNR	1.97	1.82	2.15	<0.001
Statin Use in Hospital	1.53	1.41	1.65	<0.001
**ICU Length of stay**	**IRR**	**95% Confidence Interval**		***P* Value**
**CDC Risk Factor Cohorts**				
0	Reference			
1	1.15	1.03	1.28	0.016
2	1.29	1.14	1.46	<0.001
3 or More Risk Factors	1.34	1.14	1.58	<0.001
**Age**	1.00	0.99	1.00	<0.001
**Gender**				
Female	Reference			
Male	1.09	1.04	1.14	<0.001
**Insurance**				
Medicaid as Any Payer	0.89	0.84	0.94	<0.001
**Teaching Status**				
Nonteaching Hospital	Reference			
Teaching Hospital	0.98	0.93	1.03	0.426

Table S5. Association of Risk Factors with Mortality with Broad Renal Disease Definition, ICU Utilization, ICU Length of Stay, and Hospital Length of Stay: Complete Model data

**Hospital Bed Size**				
0–99	Reference			
100–199	1.04	0.93	1.16	0.503
200–299	1.02	0.91	1.13	0.78
300–499	1.04	0.93	1.16	0.458
500 or More	1.07	0.95	1.19	0.251
**Comorbid Risk Factors**				
Chronic Lung Disease	0.93	0.87	0.99	0.016
Moderate-to-Severe Asthma	1.02	0.74	1.41	0.882
Heart Condition	1.02	0.96	1.08	0.508
Immunocompromised	0.93	0.85	1.01	0.082
Obesity	1.14	1.06	1.22	<0.001
Diabetes	0.92	0.86	0.98	0.006
Renal Disease	0.94	0.85	1.04	0.268
Liver Disease	1.09	0.99	1.20	0.074
Hypertension	1.01	0.95	1.08	0.698
DNR	1.01	0.95	1.07	0.798
Statin Use in Hospital	1.17	1.11	1.23	<0.001
Mortality	1.09	1.03	1.15	0.002
**Hospital Length of Stay**	**IRR**	**95% Confidence Interval**		***P* Value**
**CDC Risk Factor Cohorts**				
0	Reference			
1	1.16	1.12	1.21	<0.001
2	1.26	1.20	1.33	<0.001
3 or More Risk Factors	1.35	1.26	1.44	<0.001
**Age**	1.00	1.00	1.00	0.546
**Gender**				
Female	Reference			
Male	1.10	1.08	1.13	<0.001
**Insurance**				
Medicaid as Any Payer	0.98	0.96	1.00	0.028
**Teaching Status**				
Nonteaching Hospital	Reference			
Teaching Hospital	0.93	0.92	0.95	<0.001
**Hospital Bed Size**				
0–99	Reference			
100–199	1.00	0.95	1.05	0.864
200–299	1.03	0.98	1.09	0.245
300–499	1.14	1.08	1.20	<0.001
500 or More	1.13	1.07	1.19	<0.001
**Comorbid Risk Factors**				

Table S5. Association of Risk Factors with Mortality with Broad Renal Disease Definition, ICU Utilization, ICU Length of Stay, and Hospital Length of Stay: Complete Model data

Chronic Lung Disease	0.96	0.93	0.98	0.001
Moderate-to-Severe Asthma	1.10	0.96	1.25	0.162
Heart Condition	1.01	0.99	1.04	0.225
Immunocompromised	1.04	1.00	1.07	0.03
Obesity	1.15	1.12	1.19	<0.001
Diabetes	0.98	0.96	1.01	0.131
Renal Disease	1.10	1.06	1.14	<0.001
Liver Disease	1.12	1.07	1.18	<0.001
Hypertension	1.00	0.97	1.02	0.775
DNR	1.03	1.00	1.05	0.034
Statin Use in Hospital	1.14	1.11	1.16	<0.001
Mortality	1.00	0.98	1.03	0.875
